# Expanding success in the isolation of abundant marine bacteria after reduction in grazing and viral pressure and increase in nutrient availability

**DOI:** 10.1128/spectrum.00890-23

**Published:** 2023-09-25

**Authors:** Xavier Rey-Velasco, Ona Deulofeu-Capo, Isabel Sanz-Sáez, Clara Cardelús, Isabel Ferrera, Josep M. Gasol, Olga Sánchez

**Affiliations:** 1 Institut de Ciències del Mar (ICM-CSIC), Barcelona, Catalonia, Spain; 2 Instituto de Diagnóstico Ambiental y Estudios del Agua (IDAEA-CSIC), Barcelona, Catalonia, Spain; 3 Centro Oceanográfico de Málaga, Instituto Español de Oceanografía, (IEO-CSIC), Fuengirola, Málaga, Spain; 4 Departament de Genètica i Microbiologia, Facultat de Biociències, Universitat Autònoma de Barcelona, Bellaterra, Spain; University of Michigan-Ann Arbor, Ann Arbor, Michigan, USA

**Keywords:** bacterial isolates, microcosm experiments, culturability

## Abstract

Isolation of microorganisms is a useful approach to gathering knowledge about their genomic properties, physiology, and ecology, in addition to allowing the characterization of novel taxa. We performed an extensive isolation effort on samples from seawater manipulation experiments that were carried out during the four astronomical seasons in a coastal site of the northwest Mediterranean to evaluate the impact of grazing, viral mortality, resource competition reduction, and light presence/absence on bacterioplankton growth. Isolates were retrieved using two growth media, and their full 16S rRNA gene was sequenced to assess their identity and calculate their culturability across seasons and experimental conditions. A total of 1,643 isolates were obtained, mainly affiliated to the classes *Gammaproteobacteria* (44%), *Alphaproteobacteria* (26%), and *Bacteroidia* (17%). Isolates pertaining to class *Gammaproteobacteria* were the most abundant in all experiments, while *Bacteroidia* were preferentially enriched in the treatments with reduced grazing. Sixty-one isolates had a similarity below 97% to cultured taxa and are thus putatively novel. Comparison of isolate sequences with 16S rRNA gene amplicon sequences from the same samples showed that the percentage of reads corresponding to isolates was 21.4% within the whole data set, with dramatic increases in the summer virus-reduced (71%) and diluted (47%) treatments. In fact, we were able to isolate the top 10 abundant taxa in several experiments and from the whole data set. We also show that top-down and bottom-up controls differentially affect taxa in terms of culturability. Our results indicate that culturing marine bacteria using agar plates can be successful in certain ecological situations.

**IMPORTANCE:**

Bottom-up and top-down controls greatly influence marine microbial community composition and dynamics, which in turn have effects on their culturability. We isolated a high amount of heterotrophic bacterial strains from experiments where seawater environmental conditions had been manipulated and found that decreasing grazing and viral pressure as well as rising nutrient availability are key factors increasing the success in culturing marine bacteria. Our data hint at factors influencing culturability and underpin bacterial cultures as a powerful way to discover new taxa.

## INTRODUCTION

Current marine microbial ecology is largely based on culture-independent studies, yet isolation of marine microbes is still an essential process that allows performing physiological experiments and testing ecological hypotheses derived from culture-independent studies, by allowing access to whole genomes that inform about microbial metabolic capabilities and characterization of novel genes (1), through retrieval of novel taxa (2), and enabling the utilization of naturally present organisms in, e.g., bioremediation.

It is well known that most marine microbes have historically been recalcitrant to cultivation in a phenomenon labeled as the “great plate count anomaly” (3) or the “1% culturability paradigm” (4). This statement, however, has recently been disproved and we now know that higher-than-thought proportions of taxa have been cultured across biomes, in particular in the oceans (4 – 6). This is partly due to alternative culturing techniques that have been developed to increase the retrieval in culture of microorganisms from environmental samples, such as diffusion chambers (7, 8), cultivation chips (9, 10), microfluidic systems (10, 11), microencapsulation (12, 13), high-throughput culturing (14), or high-throughput dilution to extinction (HT-DTE) (15). These techniques try to overcome some of the factors that make microbes recalcitrant to cultivation, such as the requirement of specific growth factors, inorganic compounds or electron donors and acceptors which are not included in common culture media, symbiotic interdependencies which make some organisms dependent on others to be cultured, or out-competition of oligotrophs by copiotrophs (16).

Still, classic agar plates are the most economic and easy-to-implement method to culture microorganisms. This technique generally produces somehow unsatisfactory results because it is biased toward copiotrophic taxa, which normally are present in low abundances in the sea (17). However, high culturabilities have been obtained using agar plates after sudden environmentally relevant events (18), or in nutrient-rich conditions (3, 19 – 23), suggesting that changes in environmental conditions could lead to a larger culturing efficiency with this technique. In fact, low abundances and dormancy induced by poor nutrient availability (bottom-up control) are factors that limit the culturability of marine microbes (16). It is also known that copiotrophs are especially targeted by protists and viruses (top-down control) (24 – 26) and some studies propose that viral infection could be responsible for low plating efficiency (27, 28). Importantly enough, certain copiotrophs can quickly respond to changes in the environment, so that they sometimes dominate microbial communities (24, 29 – 31), and these environmental changes might be related to increased nutrient availability or reduced predator or viral mortality. We also know that light significantly determines seasonal changes in marine microbial communities and could thus also influence culturability (32 – 35).

Micro- or mesocosm experiments are a common approach to determining the effects of environmental variables on microbial abundance, activity, and diversity. They have been used, e.g., to describe the effects of phytoplankton blooms (36), oil spills (37), grazer reduction (38), or viral suppression (25) on bacterial community dynamics using molecular approaches, but so far we are not aware of culturing efforts in this type of experiments. In this study, we performed extensive bacterial isolation efforts in several manipulation experiments carried out in the four astronomical seasons that evaluated the impact of grazers, viruses, light, and resource availability on the bacterial community dynamics of the Blanes Bay Microbial Observatory (BBMO; northwest [NW] Mediterranean) using two different culture media: the standard, nutrient-rich Marine Agar 2216 (MA) and Marine Reasoner’s 2A Agar (mR2A), which has lower concentration of nutrients. Our main objectives were (i) to obtain a heterotrophic bacterial collection from Blanes Bay as diverse as possible, eventually retrieving novel taxa, (ii) to test the phylogenetic compositional differences of culturable bacteria across culture media, seasons, and experiments, and (iii) to compare the isolates with 16S rRNA gene amplicon sequencing data from the same experiments to determine the influence of environmental conditions on culturability. Thus, we isolated bacteria from initial (t_0_) and final times (t_f_) of experiments where we manipulated seawater to remove large predators in light/dark cycles (predator-reduced light [PL]) and in the dark (predator-reduced dark [PD]), to increase nutrient availability through dilution of the original bacterial community while also reducing predators in light/dark cycles (diluted light [DL]) and to add to these manipulations virus reduction in light/dark cycles (virus-reduced light [VL]). There were also unmanipulated controls for these experiments in light/dark cycles (control light [CL]) and in the dark (control dark [CD]).

## RESULTS

### Composition and diversity of the isolate collection

**Fig 1:**
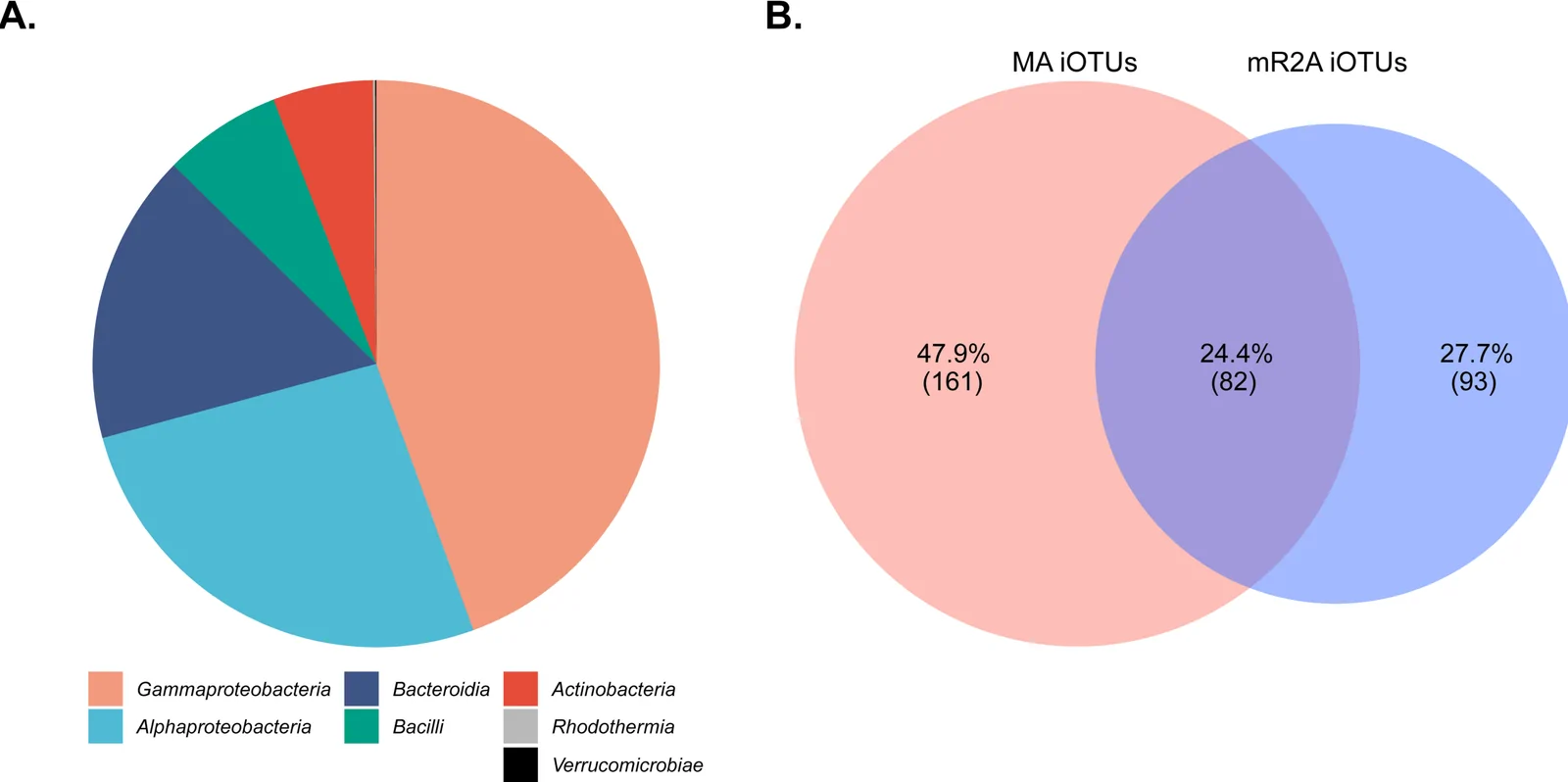
Overview of the isolate collection. (A) Pie chart showing proportional class distribution of the iOTUs. (B) Proportional Venn diagram showing similarity in iOTU composition between the two culture media used. Data calculated from the non-normalized iOTU table.

We obtained 1,643 bacterial isolates (listed in Table S1 at https://github.com/x-rv/Manuscript-2023/raw/main/Supplemental_Tables.xlsx) belonging to 5 phyla, 7 classes, 24 orders, 52 families, and 125 genera that we clustered at 99% sequence similarity into 336 isolated operational taxonomic units (iOTUs) and at 100% similarity into 715 zero-radius iOTUs (ziOTUs). The number of isolates was relatively homogeneous across culture media (816 isolates in MA, 827 isolates in mR2A), seasons and treatments, and comparatively higher in t_0_ samples altogether (Table 1). The most abundant isolates pertained to classes *Gammaproteobacteria*, *Alphaproteobacteria,* and *Bacteroidia* (Fig. 1A) and genera *Alteromonas* (360 isolates), *Limimaricola* (129 isolates), *Pseudoalteromonas* (111 isolates), *Bacillus* (78 isolates), and *Alcanivorax* (71 isolates). Interestingly, two of our isolates were affiliated to the recently described class *Rhodothermia* (39) and one to *Verrucomicrobiae*.

**TABLE 1:** Distribution of the isolates by class, season, and treatment^*b*^

Class	Season				Treatment^*a*^						
	Fall	Winter	Spring	Summer	t_0_	CL	CD	PL	PD	DL	VL
*Gammaproteobacteria*	42.6% (156)	45.9% (206)	29.5% (117)	58.1% (251)	33.8% (194)	39.6% (39)	35.9% (40)	55.5% (41)	52.0% (42)	56.2% (122)	56.2% (123)
*Alphaproteobacteria*	26.2% (41)	15.6% (43)	39.1% (155)	25.9% (112)	29.4% (169)	32.5% (44)	36.5% (45)	14.5% (24)	13.3% (19)	25.3% (46)	26.0% (45)
*Bacteroidia*	18.6% (47)	22.5% (48)	19.9% (49)	5.6% (23)	13.2% (50)	15.6% (23)	17.9% (27)	23.7% (51)	29.3% (52)	12.0% (25)	15.1% (32)
*Bacilli*	4.1% (53)	3.3% (53)	9.6% (37)	10.0% (54)	12.5% (55)	7.8% (12)	5.8% (9)	0.6% (1)	2.7% (4)	4.6% (10)	1.4% (3)
*Actinobacteria*	8.2% (29)	12.5% (40)	1.8% (7)	0.2% (1)	10.5% (56)	4.5% (7)	3.8% (6)	5.8% (10)	2.7% (4)	1.8% (4)	1.4% (3)
*Rhodothermia*	0.3% (1)	0.0% (0)	0.0% (0)	0.2% (1)	0.3% (2)	0.0% (0)	0.0% (0)	0.0% (0)	0.0% (0)	0.0% (0)	0.0% (0)
*Verrucomicrobiae*	0.0% (0)	0.2% (1)	0.0% (0)	0.0% (0)	0.2% (1)	0.0% (0)	0.0% (0)	0.0% (0)	0.0% (0)	0.0% (0)	0.0% (0)
Total	366	449	396	432	574	154	156	173	150	217	219

^*a*^ t_0_ considers all treatments at the initial time, CL to VL correspond to the final time of each treatment.

^*b*^ Relative abundances (in %) and number of isolates in brackets are presented for each class (based on SILVA classification).

The mean culturability, measured as the ratio of the concentration of plate colony counts (CFU mL^−1^) and total prokaryotic cell concentration in DAPI (4′,6-diamidino-2-phenylindole) samples (cells mL^−1^), was 0.1 ± 0.22% and it almost always increased from t_0_ to t_f_, presenting its minimum in the fall (treatment PL at t_0_, mR2A, 0.001%) and its highest values in the fall VL t_f_ (the maximum was 1.4% in MA) and summer and spring DL and VL t_f_ (Table S2 at https://github.com/x-rv/Manuscript-2023/raw/main/Supplemental_Tables.xlsx).

#### Isolate composition and diversity across culture media

In general, culturability (CFU DAPI^−1^) had slightly higher values in MA, with a mean of 0.11 ± 0.24% than in mR2A, with a mean of 0.085 ± 0.2% (Wilcoxon rank-sum test *P* = 0.07). The class composition of the isolates was highly similar in MA and mR2A, and principal coordinate analysis (PCoA) did not show culture media to explain any compositional variation (Fig. S1A at https://github.com/x-rv/Manuscript-2023/blob/main/Supplemental_Figures.pdf). However, while 51 genera were isolated with both media, 47 were only isolated in MA and 27 were unique for mR2A (Table S3 at https://github.com/x-rv/Manuscript-2023/raw/main/Supplemental_Tables.xlsx). In fact, only 82 (24.4%) iOTUs were shared among MA and mR2A (Fig. 1B).

All α-diversity indices were significantly lower in mR2A than in MA (Fig. S1B at https://github.com/x-rv/Manuscript-2023/blob/main/Supplemental_Figures.pdf). Rarefaction curves of the different media showed a similar pattern: with an equivalent sampling effort we obtained more iOTUs on MA than on mR2A and the latter was closer to reach an asymptote (Fig. S1C at https://github.com/x-rv/Manuscript-2023/blob/main/Supplemental_Figures.pdf).

#### Isolate composition and diversity across seasons

The compositional comparison between seasons at the class level is shown in Table 1. *Gammaproteobacteria* isolates had higher proportions in the summer experiment, while *Bacteroidia* were at their minimum. *Alphaproteobacteria* isolates were more numerous in the spring experiment, *Actinobacteria* were mostly isolated in the winter and the fall experiments, and the only isolate pertaining to class *Verrucomicrobiae* was obtained in the winter experiment.

The composition of the isolates at the iOTU level was significantly affected by season as shown in the PCoA followed by envfit analysis (Pr[>r] <0.001; Fig. S2A at https://github.com/x-rv/Manuscript-2023/blob/main/Supplemental_Figures.pdf). A dendrogram of the iOTU table by season using Euclidean distances indicated clustering between winter and fall, with the summer experiment clearly separated (Fig. S2B at https://github.com/x-rv/Manuscript-2023/blob/main/Supplemental_Figures.pdf).

Chao1 richness estimators and Shannon diversity indices of the isolated community were comparable in the winter and fall experiments, with lower values in the spring and especially the summer experiment. Pielou evenness and Faith’s phylogenetic diversity (FPD) were lower in the summer than in the rest of the seasons and had slightly higher values in the fall (Fig. S2C at https://github.com/x-rv/Manuscript-2023/blob/main/Supplemental_Figures.pdf). Rarefaction curves indicated that with a similar sampling effort (slightly lower in fall), the summer communities had almost reached the asymptote while the winter, with the highest iOTU number, was far from it (Fig. S2D at https://github.com/x-rv/Manuscript-2023/blob/main/Supplemental_Figures.pdf).

#### Isolate composition and diversity across treatments

To compare treatments we clustered together all t_0_ samples (untreated) into one category and compared that category to each t_f_ (treatments). All treatments except controls were enriched in *Gammaproteobacteria* compared to t_0_. *Alphaproteobacteria* relative abundances increased slightly in the control treatments and decreased when predators were reduced. *Bacteroidia* were more abundant in predator-reduced treatments compared to the rest, while *Actinobacteria* and *Bacilli* were more abundant at t_0_ (i.e., reduced their presence in all experimental treatments). The rare classes, *Rhodothermia* and *Verrucomicrobiae*, were both isolated only in t_0_ samples. On the other hand, light did not show any notable influence in isolate class composition (Table 1).

Regarding isolate similarity, the PCoA followed by *envfit* analysis showed that treatments explained the variance of iOTU composition with a significant goodness of fit (Pr[>r] <0.001) with t_0_ samples opposed to PL, PD, DL, and VL treatments, with t_f_ controls half the way between t_0_ and treatments t_f_ (Fig. S3A at https://github.com/x-rv/Manuscript-2023/blob/main/Supplemental_Figures.pdf).

Due to the high variance of the t_0_ samples and the similarity of the light and dark treatments (Table 1), we only tested the α-diversity indices at t_f_ of the light treatments. In general, isolates in DL and VL had the lowest values of the Chao1 estimator, Shannon diversity, Pielou evenness, and FPD indices while CL and PL had similarly higher values (Fig. S3B at https://github.com/x-rv/Manuscript-2023/blob/main/Supplemental_Figures.pdf). ANOVA and Tukey’s post-hoc test showed no significant differences between these values, probably due to the low number of samples in each category. Rarefaction curves (Fig. S3C and D at https://github.com/x-rv/Manuscript-2023/blob/main/Supplemental_Figures.pdf) concur with this observation: at final times, DL and VL were the treatments with the lowest number of iOTUs despite being the most sampled.

### Comparison between isolate diversity and amplicon 16S rRNA gene diversity

**Fig 2:**
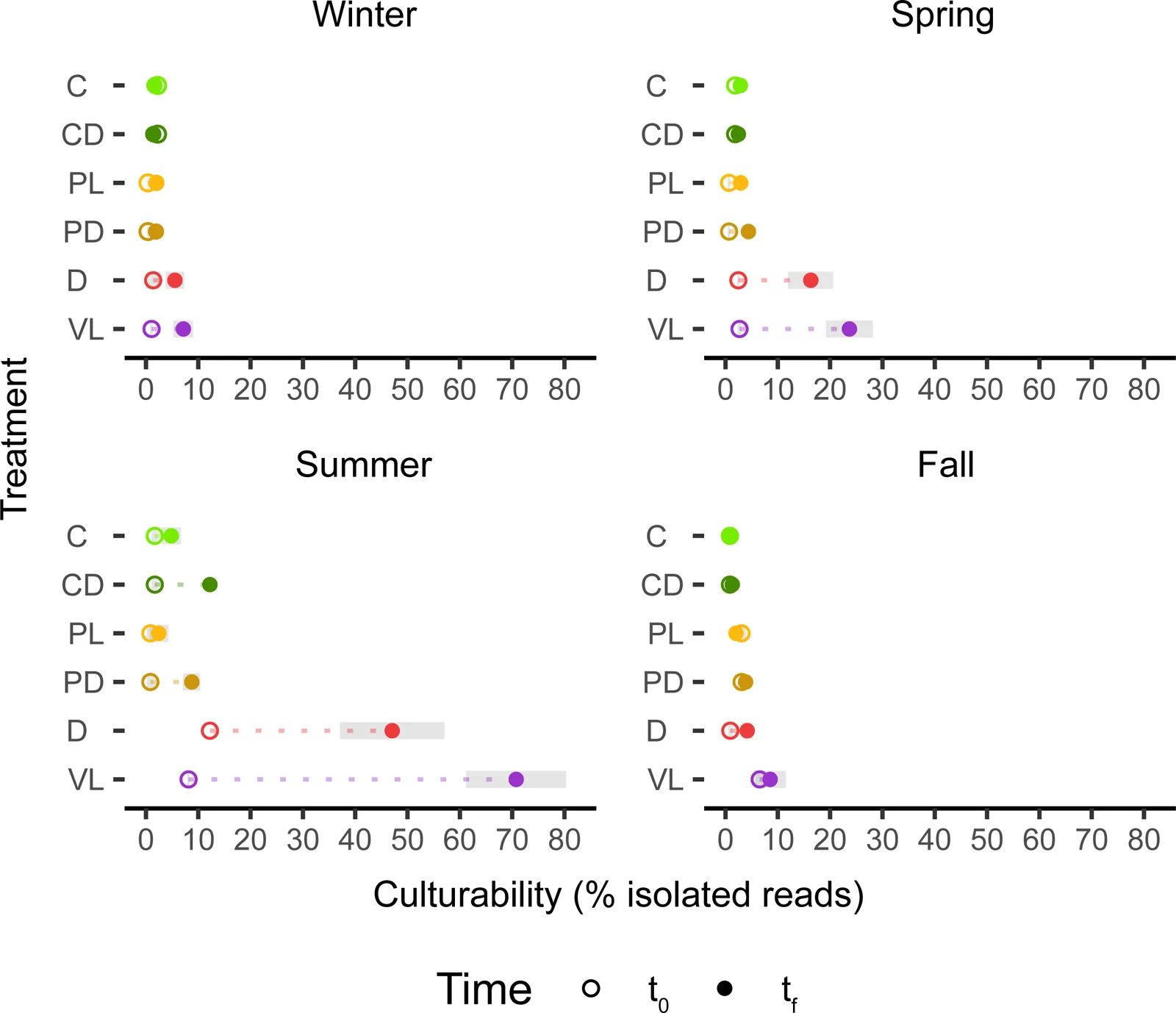
Cultured proportion of the population in each season, treatment, and time. Percentage of Illumina 16S rRNA gene reads that corresponds to isolates at 100% identity vs treatments. Hollow circles represent t_0_, full circles represent t_f_. Gray areas indicate standard deviations of replicates.

We compared the complete 16S rRNA gene sequences of our isolates with the V4-V5 region of the same gene in the amplicon sequence variants (ASVs) from the same experiments, and we found that 63.08% of our ziOTUs matched to an ASV with 100% similarity. We isolated 173 out of 4,594 ASVs (3.76%) accounting for 21.37% of the reads in the whole data set, with high variability between samples: there were lower values in t_0_ samples with a mean of 2.44 ± 2.78% than at t_f_, with a mean of 9.97 ± 16.38% (Wilcoxon rank-sum test *P* < 0.01). Importantly, this value escalated in summer and spring VL and DL treatments (Fig. 2; Table S4 at https://github.com/x-rv/Manuscript-2023/raw/main/Supplemental_Tables.xlsx displays a complete list), reaching as high as 70.76% cultured reads in the summer VL treatment and 47.09% in spring VL. It also increased notably in the summer CD treatment, while in the fall and winter VL treatment the values were more modest but still higher than at t_0_.

**Fig 3:**
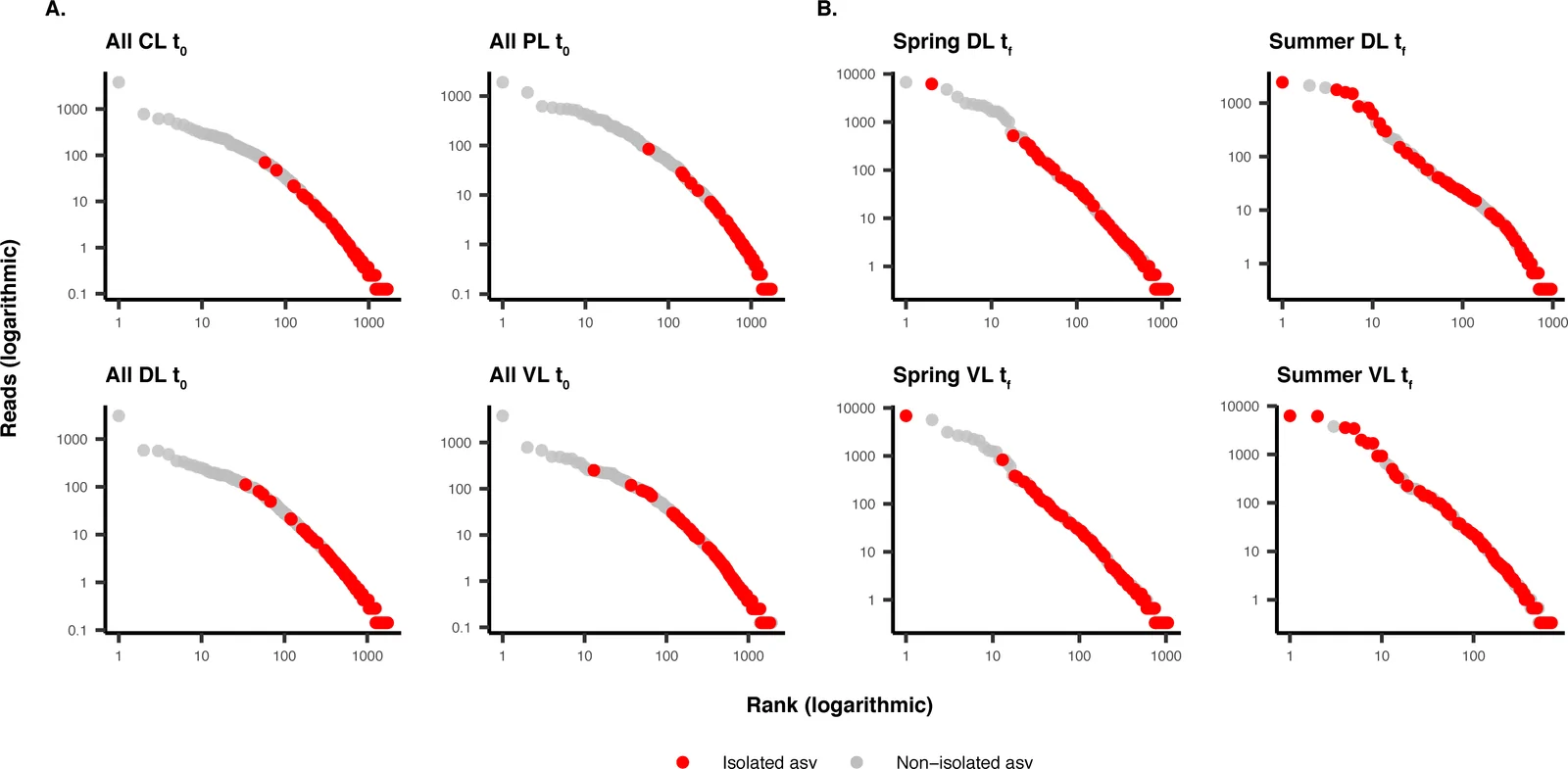
Selected rank abundance plots based on 16S rRNA gene amplicon sequencing (region V4-V5) with indication of the cultured organisms that match the sequences (in red). The non-isolated ASVs are represented in gray. (A) Examples where we only isolated rare taxa. All seasons are considered. (B) Summer and spring samples where we isolated dominant taxa.

While in most samples we cultured taxa pertaining to the rare biosphere (Fig. 3A; Fig. S4 at https://github.com/x-rv/Manuscript-2023/blob/main/Supplemental_Figures.pdf), in some we isolated very relevant ASVs in terms of abundance (Fig. 3B). We cultivated organisms that were 100% equal to almost all the top rank taxa detected by amplicon sequencing of the V4-5 region of 16S rRNA gene in the summer experiment VL and DL t_f_ treatments, affiliated to genera *Alteromonas*, *Vibrio,* and *Limimaricola*. Interestingly, we also isolated ranks six and eight of summer CD treatment at t_f_ that pertained again to genus *Alteromonas*. In the spring experiment, we cultured the first ASV of VL treatment t_f_ and the second from DL treatment t_f_, affiliating to genus *Nereida*. In the fall and winter experiments, we obtained more modest but still notable results from treatment VL t_f_. Ranks four, five, and eight (genera *Alteromonas*, *Halomonas,* and *Nereida*) were isolated in the fall experiment while in the winter experiment we cultured organisms 100% identical to ranks seven, eight, nine, and ten (genera *Vibrio*, *Polaribacter*, *Lentibacter,* and *Colwellia*). We isolated *Pseudoalteromonas*, which was in rank eight of winter DL t_f_, and *Tenacibaculum*, which appeared in important positions at t_f_ of the winter, fall, and spring VL experiments (ranks 14, 16, and 19, respectively). Surprisingly, there were some t_0_ samples from where some isolates were identical to relevant taxa: the summer experiment, treatment DL t_0_ (ranks three and four, *Limimaricola* and *Palleronia*), and the fall experiment VL t_0_ (rank four, *Halomona*s). Importantly, we isolated organisms 100% identical to ranks three, six, seven, and nine of the whole data set pertaining to genera *Alteromonas* and *Vibrio*. Ranks, mean abundances, and closest neighbors of all the ASVs with identical cultured organisms can be found in Table S5 (at https://github.com/x-rv/Manuscript-2023/raw/main/Supplemental_Tables.xlsx) for each season and treatment and Table S6 (at https://github.com/x-rv/Manuscript-2023/raw/main/Supplemental_Tables.xlsx) for the data set as a whole.

**Fig 4:**
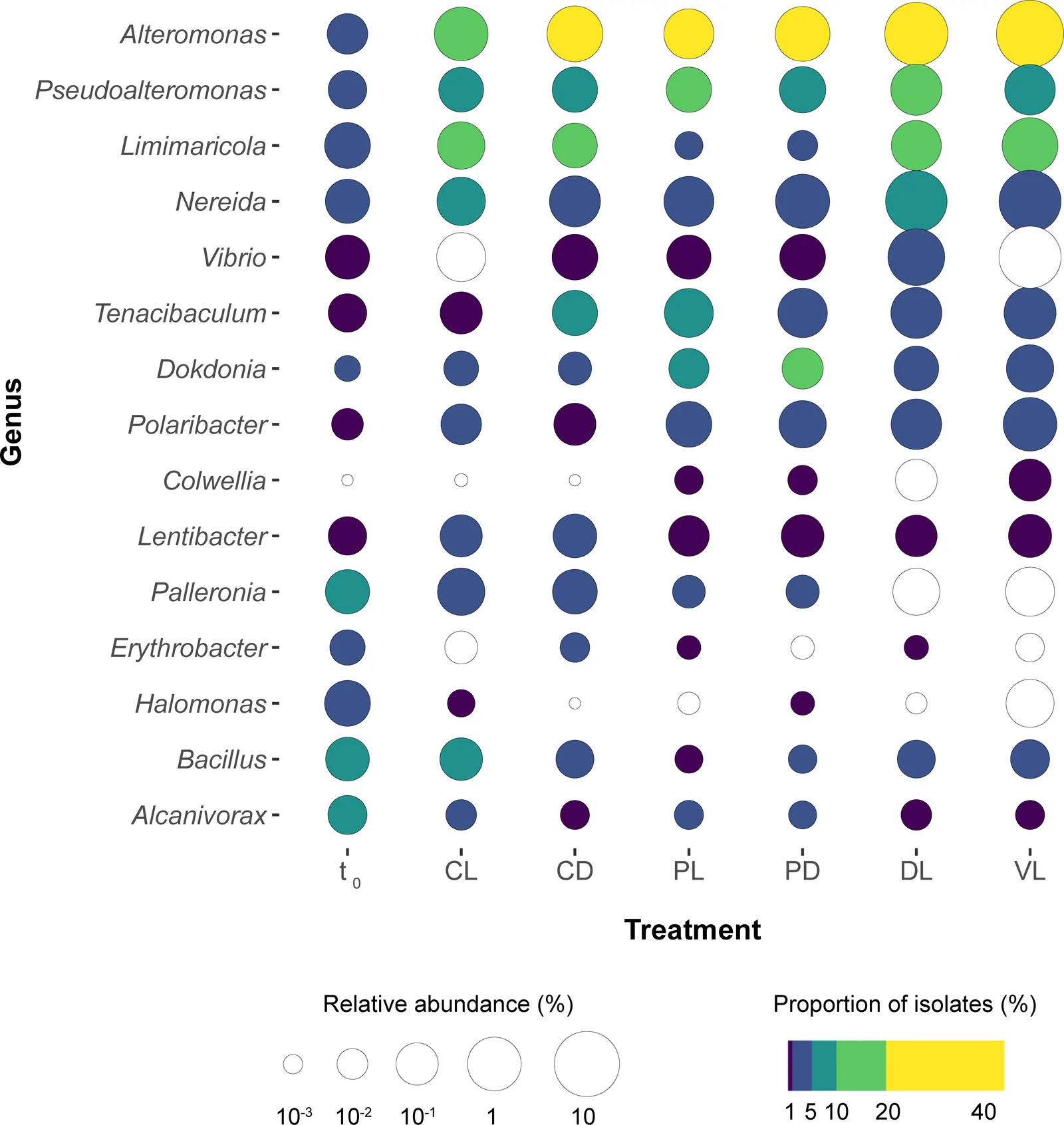
Culturability (proportion of isolates) and relative abundances of relevant genera in this study in the different treatments. White color means that the particular genus was not isolated in that treatment. Category t_0_ incorporates all treatments at the initial time; CL to VL correspond to the final times of each treatment.

To decipher the link between culturability and the manipulations done in this study, we integrated the information of *in situ* ASV relative abundances (Table S5 at https://github.com/x-rv/Manuscript-2023/raw/main/Supplemental_Tables.xlsx) and the frequency of isolation for the most relevant genera (those which reached high abundances in the treatments, and the 10 most cultured) in Fig. 4. This shows that especially *Alteromonas* were very frequently cultured in all the t_f_ of the experiments, coupled with an increase in their relative abundances. A similar but slightly less strong trend could be seen for *Pseudoalteromonas*. Other genera that were more culturable and abundant in some or all of the manipulations were *Limimaricola*, *Nereida*, *Vibrio*, *Tenacibaculum*, *Dokdonia*, *Polaribacter*, *Colwellia,* and *Lentibacter*. All of them showed higher culturabilities in specific treatments, for example, *Limimaricola* were more culturable in the control, DL, and VL treatments, which correlated with their abundances; *Vibrio* were more culturable in DL while in VL, despite being more abundant, were not cultured; *Dokdonia* preferred predator-reduced treatments despite being more abundant in the stronger manipulations (DL and VL) and *Colwellia* were only cultured in some of the manipulations. On the contrary, *Palleronia*, *Erythrobacter*, *Halomonas*, *Bacillus,* and *Alcanivorax* were more culturable in t_0_ samples, despite sometimes being more abundant at the end of the manipulations.

### Novelty of the isolate collection

**Fig 5:**
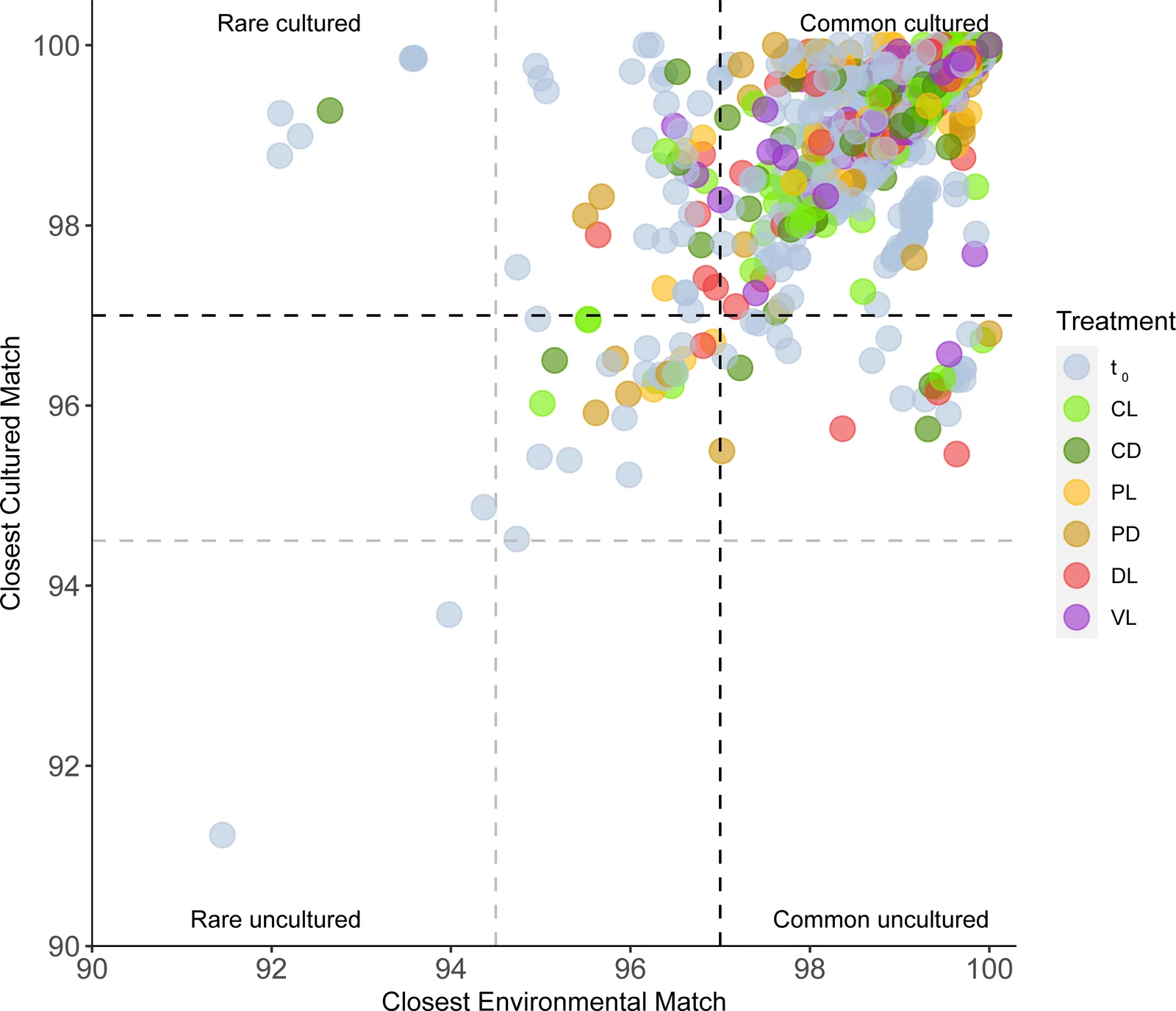
Percentage similarity between the CCM and the CEM of isolate 16S rRNA gene sequences. Horizontal and vertical lines represent the typical cut-off values of 97% (black dashed lines) commonly used for species delineation, and the cut-off values of 94.5% (gray dashed lines) used for genera delineation. Colored by treatment. Category t_0_ incorporates all treatments at the initial time; CL to VL correspond to the final times of each treatment.

To test the novelty of our collection, we plotted the closest environmental match (CEM) vs the closest cultured match (CCM) of our isolates (Fig. 5). This presents 61 isolates (3.7% of total isolates) that belong to 39 iOTUs (11.6% of total iOTUs) and 48 ziOTUs (6.7% of total ziOTUs) with less than 97% similarity with their CCM (i.e., they correspond to taxa that have never been cultured). Thirty isolates (1.8%) scored between 94.5% and 97% similarity with both CCM and CEM, which could represent 19 genuinely novel species, as was revealed by clustering them to 97% similarity. Moreover, two isolates had less than 94.5% similarity with neither their CCM nor CEM*,* and thus they could represent two novel genera (40).

**Fig 6:**
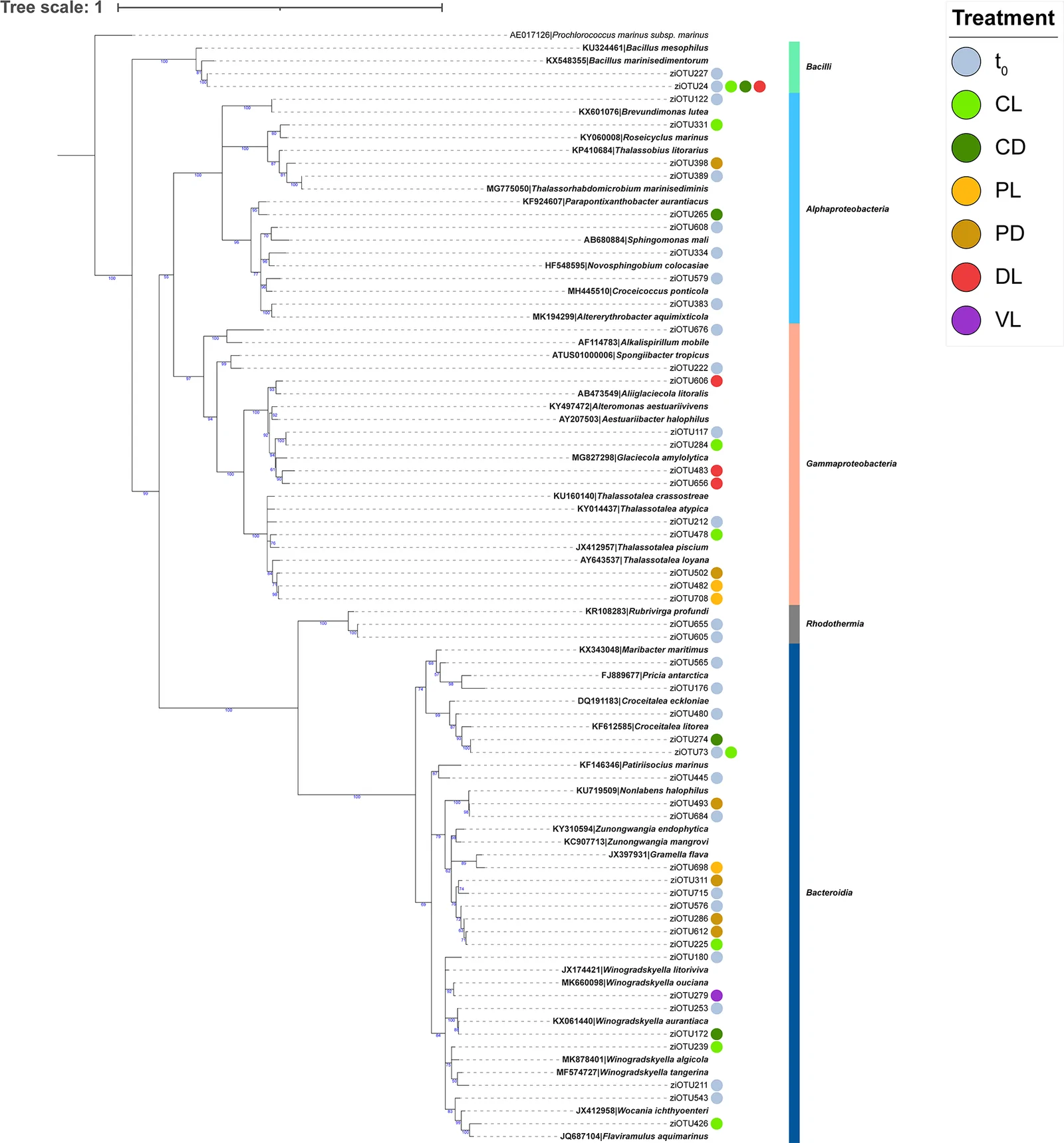
Phylogeny of putative novel isolates. Phylogenetic tree includes 48 ziOTUs with <97% similarity to their CCM in the RDP database and their closest match in the SILVA Living Tree Project (in bold). The numbers in the nodes represent bootstrap coefficients calculated from 1,450 replicates. Non-supported branches (bootstrap coefficients below 50%) were collapsed. Colored circles represent the treatments from where the ziOTUs were isolated. Classes to which the different taxa pertain are also indicated. Category t_0_ incorporates all treatments at the initial time; CL to VL correspond to the final times of each treatment.

To examine the phylogenetic placement of the novel strains, we constructed a phylogenetic tree with the putative novel ziOTUs (Fig. 6), which indicates that novel isolates pertained mostly to classes *Bacteroidia, Gammaproteobacteria, and Alphaproteobacteria*. There were three ziOTUs affiliated to class *Bacilli*, and two ziOTUs to class *Rhodotermia*, which interestingly were the only ones from this class in the whole collection. Novel isolates were mostly cultured in MA (35 ziOTUs in MA, 13 in mR2A) and distributed similarly between seasons. Most novel ziOTUs were obtained from t_0_ samples (twenty-six) with some in control treatments (eleven), predator-reduced (nine), and less frequently DL (four) and VL (one) treatments.

## DISCUSSION

We have obtained an extensive collection of 1,643 isolates from different manipulation experiments that were carried out in the four astronomical seasons, using two distinct culture media. Overall, the phylogenetic distribution of our collection at the class level (Table 1) is similar to that obtained in other studies made in the same sampling site (41). It is noteworthy, though, that classes *Rhodotermia* and *Verrucomicrobiae* had never been isolated in the BBMO, though the latter had been detected by culture-independent approaches (e.g., reference 41). In addition to this general picture, one of the aims of this study was to test differences in the phylogenetic composition and diversity of our isolates across culture media, seasons, and treatments.

Culture medium MA has a higher organic matter concentration (5 g L^−1^ proteose peptone, 1 g L^−1^ yeast extract, total 6 g L^−1^ organic matter) than mR2A, while the latter presents a more diverse composition in terms of carbon sources (0.5 g L^−1^ proteose peptone, 0.5 g L^−1^ casamino acids, 0.5 g L^−1^ yeast extract, 0.5 g L^−1^ dextrose, total 2.5 g L^−1^ organic matter); therefore, the use of these two media aimed to increase the diversity of the obtained heterotrophic isolates. This goal was achieved, as the proportion of shared genera (Table S3 at https://github.com/x-rv/Manuscript-2023/raw/main/Supplemental_Tables.xlsx) and iOTUs (Fig. 1B) across culture media was low. We expected to obtain higher CFU mL^−1^ in mR2A considering that previous studies have shown that more oligotrophic culture media resulted in better culturabilities than standard rich media (14, 42). However, in this study mR2A yielded slightly lower values than MA. This could have happened because mR2A is not oligotrophic (i.e., low-nutrient) enough to manifest this effect, and after all it still contains 2.5 g L^−1^ organic matter. Probably, using a more nutrient-poor medium such as the modified seawater medium (SW) (14, 43) could have led to the isolation of other taxonomic groups adapted to the low nutrient availability observed in the environment.

The isolate composition varied significantly across seasons: fall and winter were the most similar seasons and summer the most distant (Fig. S2B at https://github.com/x-rv/Manuscript-2023/blob/main/Supplemental_Figures.pdf). This is the exact same trend that was observed for the structure of the environmental bacterial communities in the BBMO as determined by DGGE in Alonso-Sáez et al. (34), and by 16S rRNA gene amplicon sequencing (44). Our isolates reached their lowest α-diversity in summer with higher values in the spring and, especially, in the fall and winter (Fig. S2C at https://github.com/x-rv/Manuscript-2023/blob/main/Supplemental_Figures.pdf). Other studies carried out in the BBMO using molecular methodologies have shown similar tendencies (34, 44). While the seasonality of microbial communities in BBMO has been broadly described (32, 34, 44 – 46), this is the first evidence of seasonality in the culturable bacteria fraction of the BBMO community.

The dominance of Gammaproteobacterial isolates in all treatments except the controls (Table 1) correlates with CARD-FISH relative abundances in these experiments (32) which were seen to be high for this class and especially for *Alteromonadaceae* in the mentioned samples. Likewise, the isolation of comparatively more taxa affiliating to class *Bacteroidia* in the PL and PD treatments (Table 1) is in accordance with the same data: CARD-FISH abundance corresponding to this class peaked especially in the winter and spring PL and PD treatments (32). The PCoA by treatment (Fig. S3A at https://github.com/x-rv/Manuscript-2023/blob/main/Supplemental_Figures.pdf) suggests that while the sole fact of confining seawater in bottles caused a change in the composition of culturable bacteria, a known effect that has already been reported (47, 48), treatments that implied a real manipulation of environmental conditions (PL, PD, DL, and VL) had by themselves a notable effect in changing the composition of the culturable bacterial community. This compositional change did not affect α-diversity in PL and PD; however, it had an effect in DL and VL treatments (Fig. S3B at https://github.com/x-rv/Manuscript-2023/blob/main/Supplemental_Figures.pdf), where α-diversity decreased, suggesting that these treatments favored a narrow set of culturable bacteria over the other treatments. In the microcosm experiments performed by Teira et al. (38), the α-diversity indices were reduced when reducing grazer pressure (equivalent to the PL treatment here), but here it did so only with deeper manipulations (DL and VL). While Teira et al. performed their experiments in offshore waters in different oceans (Atlantic, Pacific, and Indian) that were analyzed with 16S rRNA gene amplicon sequencing, we observed a similar trend in the coastal Mediterranean sea.

When comparing the 16S rRNA gene sequences of isolates and ASVs (V4-V5 region), we observed that 63.08% of our ziOTUs were identical to an ASV and 3.76% of all ASVs were represented by isolates. It is common to find similar or lower proportions of isolates represented by sequences detected using molecular approaches (14, 22, 41, 49, 50). This is because taxa recovered by isolation usually belong to the “rare biosphere” of a given environment, while molecular techniques retrieve preferentially the relatively more abundant bacteria (17, 51). The low proportion of ASVs represented by isolates is actually similar to the value found in other studies (23, 50).

In this study we demonstrate that, using agar plates, it is possible to isolate dominant heterotrophic marine bacteria that represent a significant proportion of the population. In fact, our isolates accounted for 21.37% of the total reads and this percentage was higher in certain samples, reaching up to 70.76% (in the summer experiment, treatment VL, Fig. 2; Table S4 at https://github.com/x-rv/Manuscript-2023/raw/main/Supplemental_Tables.xlsx). Studies that compare isolates with sequencing data are scarce and they usually do not provide culturability values. Among the ones that made available these data, Sanz-Sáez et al. (22) obtained a mean of 0.3–1.1% with 7.8% as the maximum proportion of reads matching with isolates in samples from global oceans at different depths. Wang et al. (23) isolated 45% of their whole 3-sample data set from marine sediments, yet they applied a 97% similarity threshold to consider an OTU as cultured and thus, this number was likely an overestimation in comparison with our approach. Alejandre-Colomo et al. (14) obtained a mean of 5.75 ± 2.99% cultured reads during a phytoplankton bloom in the North Sea, with a maximum of 11.51% in one sample (calculated from their Table S4 at https://github.com/x-rv/Manuscript-2023/raw/main/Supplemental_Tables.xlsx); however, they could not isolate any of the most abundant taxa. Sanz-Sáez et al. (52) obtained isolates representing up to 45% of the reads in the bathypelagic particle-associated fraction and isolated the most abundant ASVs in this fraction and ocean depth, reinforcing the idea that isolation of heterotrophic bacteria can be successful in certain environments or ecological situations.

Other studies that used alternative culturing techniques in natural samples, mainly HT-DTE, have obtained similar results in terms of the proportion of isolates matching to sequences seen through culture-independent approaches, but much better results in terms of culturability. While we isolated 3.76% of the total ASVs, Henson et al. (15) obtained 5% of them in coastal samples, although they used a 99% identity threshold. Bartelme et al. (53) obtained 20% culturability from soil samples in their HT-DTE experiments, with 11% of the total community isolated at 97% identity, while Yang et al. (54) and Connon et al. (55) obtained, respectively, 12% and 14% culturability in surface waters. Interestingly, Kim et al. (56) reached up to 34.6% culturability in a freshwater sample with a modified HT-DTE protocol supplemented with catalase. Other studies have isolated top-abundant taxa with flow sorting combined with diverse culture media in soil samples (57) or a combination of diffusion chambers and HT-DTE in coastal waters (58). All the above-mentioned studies obtained higher culturability values compared to our study, in which we reached a maximum of 1.4% (Table S2 at https://github.com/x-rv/Manuscript-2023/raw/main/Supplemental_Tables.xlsx). Thus, it seems that techniques designed for culturing oligotrophs are best to isolate the microbial majority in non-experimentally modified seawater conditions.

In general, the DL and VL treatments were the ones with the highest proportions of reads in the 16S rRNA gene amplicon sequencing data set matching exactly with an isolate (Fig. 2). The ASVs that accounted for high percentages of reads in these treatments and were 100% identical to isolates in this collection (Tables S5 and S6 at https://github.com/x-rv/Manuscript-2023/raw/main/Supplemental_Tables.xlsx) mostly pertained to genera *Alteromonas, Vibrio, Limimaricola,* and *Nereida*, fast-growing r-strategists that have commonly also been isolated in other studies (14, 22, 41), and affiliate to phylogenetic groups which have been reported to actively respond to the increase of resource availability in the environment during events like phytoplankton blooms (30, 36, 59 – 61). In this case, treatments DL and VL provided increased resource availability. In view of these data, it looks like the high proportions of reads affiliating to cultured taxa in this study are caused by the effects of the experimental manipulations, especially the increase in nutrient availability in DL and VL, that favored the growth of copiotrophic taxa which are normally found in the environment as part of the rare biosphere (17) but can become dominant under certain conditions, such as the ones created in the experiments (25, 31, 62, 63). This also suggests that r-strategists are more strongly limited by bottom-up (resources) controls than by top-down (grazers and viruses) factors.

The effect of experimental treatments in terms of isolation success (i.e., having cultured top-rank taxa and high percentages of reads affiliating to isolates) appears to be markedly different between seasons. Summer was clearly the most successful season, followed by spring, while in the fall and winter experiments, the various treatments had very weak effects (Fig. 2; Fig. S4 at https://github.com/x-rv/Manuscript-2023/blob/main/Supplemental_Figures.pdf). This could partially be explained by looking at the identities of the most abundant ASVs that we did not isolate in this study. In the DL and VL treatments (t_f_), the most abundant uncultured taxa accounting for a high proportion of fall, winter, and spring reads affiliated to a total of 12 species in the NCBI 16S rRNA database (Table S7 at https://github.com/x-rv/Manuscript-2023/raw/main/Supplemental_Tables.xlsx) and, except for *Donghicola eburneus*, they had been isolated in different conditions (culture media or temperature) and/or from other environments different than the ones used in this study (see references in Table S7 at https://github.com/x-rv/Manuscript-2023/raw/main/Supplemental_Tables.xlsx). It is also important to note that *in situ* temperatures in summer (21.3°C [32]) and fall (19.5°C [32]) were the closest to the one used in this study (RT; 20–25°C). All this suggests that isolation success was higher in summer and spring because dominant taxa in these seasons were easier to culture with our methods, especially our used culture media.

We isolated low proportions of the community in t_0_ samples (Fig. 2) and, except for some specific treatments and seasons, we did not isolate dominant taxa in terms of abundance (Fig. 3A). Most of the top-abundant ASVs in untreated samples pertain to oligotrophic genera such as *Prochlorococcus* or *Pelagibacter* that cannot be isolated in agar plates using nutrient-rich media. On the contrary, they have been isolated with methods such as HT-DTE (15) or the use of complex media (64). A taxon affiliating to species *Aquibacter zeaxanthinfaciens* was among the top ASVs but, in contrast to this study, had been isolated using the DTE plating method (65). This supports the idea that a change in the microbial community caused by the manipulations selected more readily culturable microorganisms (at the final times), so taxa that are normally abundant in the environment (the ones in initial times) are missed by our approach.

It is well known that PCR-based sequencing overrepresents the abundances of bacteria with multiple 16S rRNA gene copies (66). In fact, all our isolates that accounted for high numbers of reads are known to harbor numerous copies of the ribosomal operon according to the rrnDB database (67); therefore, it is likely that our proportions of reads corresponding to isolates are overestimated. For this reason, we generated a revised ASV abundance table by dividing each ASV read value by the number of 16S rRNA gene copies obtained from rrnDB (67) according to their class assignment, and recalculated the proportions of cultured reads in relation to these “corrected” values. With “correction,” the mean proportions of reads corresponding to isolates changed from 6.20 ± 12.23% to 5.48 ± 10.70% and the raw maximum of 70.76% decreased to 59.66% (Table S4 at https://github.com/x-rv/Manuscript-2023/raw/main/Supplemental_Tables.xlsx). These estimations would only slightly reduce our isolation success, showing nonetheless the same trend.

We wanted to explore how the different treatments influenced the culturability of genera that were relevant in this study, also taking into account their abundances as determined by amplicon sequencing. We know that when using rich culture media, the culturability of a given taxon is determined by its ability to grow faster than potential competitors (16). Thus, the organisms with increased culturability in a given experiment would be the most adapted to grow on the solid media used and the particular experimental conditions. It is shown in Fig. 4 that the genus most benefited by the manipulations in terms of culturability was *Alteromonas*, which developed better than all the other taxa even in the control treatments, in accordance with its well-known opportunistic behavior (47). The culturability of *Pseudoalteromonas* also increased in all manipulations, but its higher isolation in PL and DL indicates that this genus might be limited both by predators and nutrient availability, and that light could be beneficial for its cultivation. Some species of *Pseudoalteromonas* are photoheterotrophs and, in fact, they have been seen to have higher abundances in the light compared to darkness (68). *Limimaricola* seems to be an opportunistic genus scarcely influenced by grazing, given that its abundance and culturability did not increase in the PL and PD treatments. The comparably higher cultivation of genera *Nereida* and *Vibrio* in the DL treatment suggests that they are especially subject to nutrient availability. Importantly, *Vibrio* was not isolated in the VL treatment despite being more abundant than in the rest of the experiments, indicating that the presence of virus might favor its growth on solid media, or that other groups are more sensitive to viral lysis. *Tenacibaculum* and *Dokdonia* were clearly benefited by predator reduction while *Polaribacter* seems to develop well in all conditions. The genus *Colwellia* was only isolated in the manipulations, where it also increased in relative abundance, suggesting poor cultivation in unmanipulated conditions and a high influence of predation and viral lysis. The slight increase of *Lentibacter* isolation in controls implies that this genus has an opportunistic behavior, but it does not develop well on plates when manipulations are stronger. Finally, genera *Palleronia*, *Erythrobacter*, *Halomonas*, *Bacillus,* and *Alcanivorax* were more easily isolated in initial times despite sometimes being more abundant in the manipulations, suggesting that they are less adapted to grow on plates than other taxa when conditions are favorable to copiotrophic growth. Copiotrophs are known to be especially subject to grazer control, influenced by nutrient availability and targeted by viruses (24 – 26); however, we have shown here that each taxonomic group seem to be differentially influenced by these factors in terms of culturability (using agar plates) and how some genera are more competitive (i.e., in terms of developing on the plates) than others when limitations are reduced.

The other main outcome of this study is that our isolation effort resulted in a high proportion (3.7% of all isolates) of putative novel taxa (Fig. 5). This novelty is comparatively higher than that found in other studies focused on isolation, such as the one from Ma et al. (69), in which 1.9% isolates represented potential novel species in samples from deep-sea water and sediments from the Mariana Trench using six culture media, or the 0.2% isolates representing novel genera in samples from different depths across the global ocean plankton using one culture medium (22). This could be explained as a result of the extensive isolation carried out from a single sample from one site and also by the colony-picking strategy seeking different morphologies. It is noteworthy that the rarefaction curve of the study (Fig. S1D at https://github.com/x-rv/Manuscript-2023/blob/main/Supplemental_Figures.pdf) had not reached an asymptote, implying that a greater isolation effort could have resulted in the discovery of more novel isolates. In fact, higher numbers of potential novel isolates have been obtained with a remarkable culturing effort using high-throughput isolation techniques (14). The novelty obtained in this study was not equally relevant across culture media as more novel ziOTUs were found in MA, which is logical if we attend to the alpha-diversity values (Fig. S1B at https://github.com/x-rv/Manuscript-2023/blob/main/Supplemental_Figures.pdf) and rarefaction curves (Fig. S1C at https://github.com/x-rv/Manuscript-2023/blob/main/Supplemental_Figures.pdf): MA was less selective than mR2A and permitted the culturability of a wider range of bacteria. It is also noteworthy that most putative novel taxa were isolated from t_0_ and control treatments. Taking into account that the sampling effort at t_0_ (considering all samples) was 3–4 times higher than that of the treatments at t_f_ (Table 1), it is not surprising to find more novel taxa there. Also, it is coherent that the frequency of isolation of novel taxa diminishes as the manipulation of environmental conditions is stronger, since it implies the selection of a smaller set of taxa. Alpha-diversity (Fig. S3B at https://github.com/x-rv/Manuscript-2023/blob/main/Supplemental_Figures.pdf) and rarefaction curves (Fig. S3C and D at https://github.com/x-rv/Manuscript-2023/blob/main/Supplemental_Figures.pdf) concur with this idea: among final times, less diversity was found in the more manipulated treatments (DL and VL).

We conclude that our extensive isolation effort applied to experimental manipulation experiments resulted in the isolation of responsive taxa corresponding to exceptionally high proportions of the microbial population, although they were mainly common copiotrophs. We are far from being able to obtain all the natural environment microorganisms in culture, but our data show that we can find a reasonable number of the bacteria responding to manipulations, indicating that culturability is highly influenced by environmental factors, especially resource availability, grazing, and viral lysis.

Overall, our results point to environmental conditions as key factors influencing isolation success. Also, culturing microorganisms with traditional methods proves useful to discover novel taxa and isolate those members of the community that are most abundant under certain environmental conditions.

## MATERIALS AND METHODS

### Origin of samples

Surface seawater samples were collected from the BBMO in the NW Mediterranean (41°40′N, 2°48′E), about 70 km north of Barcelona, and approximately 1 km offshore. Samples were collected on the four astronomical seasons: winter (21 February 2017), spring (26 April 2017), summer (5 July 2017), and fall (7 November 2017) and water was filtered *in situ* through a 200-µm mesh and transported to the laboratory within 2 h.

### Manipulation experiments

Six experimental treatments were set up the following day for each season as described in Sánchez et al. (32). Briefly, the treatments consisted of the following: (i) unfiltered seawater in light/dark cycles (CL) and in the dark (CD), (ii) seawater prefiltered through a 1-µm filter to remove large predators while preserving most bacteria in light/dark cycles (PL) and in the dark (PD), (iii) unfiltered seawater diluted 1/4 with 0.2-μm-filtered seawater to reduce predators and increase nutrient availability for bacteria in light/dark cycles (DL), and (iv) unfiltered seawater diluted 1/4 with 30-kDa-filtered seawater to reduce predators, viruses, and to increase nutrient availability, in light/dark cycles (VL). The different treatments were incubated in triplicated 9 L Nalgene bottles for 48 h at *in situ* temperature (see Table 1 in Sánchez et al. [32]) in a water bath with circulating seawater. Light treatments were limited to photosynthetically active radiation, and dark treatments were covered with several layers of dark plastic.

Samples were taken for bacterial isolation at the same time as samples for inorganic nutrient concentration and other ancillary data (reported in reference 32), DAPI total counts, CARD-FISH, and 16S rRNA gene amplicon sequencing. Samples were obtained at times 0 h, 12 h, 24 h, and 36 h in summer and winter or 48 h in the fall and spring experiments. For isolation, 1 mL seawater subsamples were mixed with 75 µL dimethyl sulfoxide (DMSO) in cryovials that were stored at −80°C in triplicates. In the winter experiment, samples for isolation were not taken at 0 h; therefore, 12 h after the start of the experiments was our initial time for isolation in this season.

### Community DNA extraction and sequencing

For 16S rRNA gene amplicon sequencing, samples were prefiltered through a 20-µm mesh to remove large particles, and microbial biomass was concentrated onto 0.2-µm-polycarbonate filters using a peristaltic pump. About 2–4 L were filtered from each replicate of all treatments. We extracted the DNA from the filters as described in Massana et al. (70), and then purified and concentrated it using Amicon 100 columns (Millipore) and quantified it in a NanoDrop-1000 spectrophotometer (Thermo Scientific). We stored the DNA at −80°C and an aliquot from each sample was used for sequencing using a MiSeq sequencer (2 × 250 bp, Illumina). A first run was sent to the Integrated Microbiome Resource (Halifax, NS, Canada; https://imr.bio) and a second run was sent to the Research and Testing Laboratory (Lubbock, TX, USA; http://rtlgenomics.com/) in order to improve the quality of some of the samples. Primers 515F-Y (5′-GTG YCAG CMG CCG CGG TAA) and 926R (5′-CCG YCA ATT YMT TTR AGT TT) from Parada et al. (71) were used to amplify the V4-V5 regions of the 16S rRNA gene.

### Amplicon sequencing data processing and taxonomic classification

We obtained two different runs of sequences that needed to be processed separately. Initially, we used cutadapt (72) to trim primers. Then, ASVs were obtained running DADA2 1.18.0 version (73), which consisted of different steps. The qscore plots were used to inspect the quality of our samples and decide where to trim. The subsequent step was running the DADA2 process using the pool method to increase sensitivity to sequences that might be present at very low frequencies in multiple samples. Finally, we merged the two runs and removed chimeras keeping 82.7% ± 0.1 sequences per sample and obtaining the final ASV table. Taxonomic assignation was performed with DECIPHER 2.16.1 version (74) at 60% confidence aligning against the SILVA database (SILVA_SSU_r138_2019.RData). Four samples with <5,000 reads were discarded, keeping a total of 308 samples for further analyses.

### Isolation of bacteria

Initial experiment sampling times (t_0_) and final (t_f_) of dark/light treatments from each season were used for isolation. Samples from 0 h of dark treatments from the control and predator-reduced experiments were not employed for this purpose, since at that point there had not been time for the light regime to cause an effect; therefore, the total number of samples was 42. Two different solid culture media were used, MA (Difco) and mR2A, which consisted of R2A Agar (Difco) prepared in Milli-Q water with 40 g L^−1^ Sea Salts (Sigma) (75), pH adjusted to 7.6. For liquid cultures, Marine Broth 2216 (Difco) and mR2A Broth prepared with R2A Broth (Neogen) in Milli-Q water with 40 g L^−1^ Sea Salts (Sigma) were used.

Aliquots of 100 µL of undiluted, 1:10, and 1:100 diluted seawater were spread in triplicates on agar plates and incubated at room temperature (20–25°C) until no more colonies appeared (maximum 30 days). Colonies with different morphologies were selected from each sample and streaked on new agar plates in order to obtain pure cultures. These cultures were then transferred to liquid medium, and after turbidity was detected, 100 µL from the suspensions were kept at −20°C for DNA extraction, while the rest was stored in 25% glycerol in cryovials at −80°C. Culturability was calculated as the ratio between the concentration of CFU mL^−1^ in agar plates and the total concentration of cells obtained from DAPI counts (DAPI mL^−1^, data obtained from Sánchez et al. [32]).

### PCR amplification and sequencing of isolates

Genomic DNA was extracted from 100 µL of liquid cultures incubated 10 min at 99°C in a thermal cycler and 10 min at −20°C for three times. These extractions were used to PCR-amplify the nearly complete 16S rRNA gene with primers 27Fmod (5′-AGR GTT TGA TCM TGG CTC AG-3′) and 1492Rmod (5′-TAC GGY TAC CTT GTT AYG ACT T-3′) from Page et al. (76). Each PCR reaction was composed of 32.75 µL Milli-Q water, 10 µL 5× Green GoTaq Reaction Buffer (Promega), 1 µL dNTPs mix (each deoxynucleotide at 10 mM), 2 µL of each primer (10 µM), 0.25 µL GoTaq DNA Polymerase (Promega), and 2 µL of extracted DNA. When faint or no bands were observed on agarose gel electrophoresis following PCR, DNA extraction was repeated with DNeasy Blood&Tissue Kit (Qiagen) following the manufacturer’s recommendations. Purification and OneShot Sanger sequencing of PCR products was carried out by Genoscreen (Lille, France) with the two above-mentioned primers.

### Isolates data processing and taxonomic classification

The sequences were manually quality-checked, trimmed, and assembled with Geneious software v.2022.0.1 (77). The UCLUST algorithm from USEARCH software (78) was used to cluster sequences at 99% similarity (79) in order to infer iOTUs. These iOTUs were used for all the subsequent analyses except to compare isolates and ASV sequences and to compute a phylogenetic tree to describe putative novel isolates, for which a 100% identity clustering was performed to define ziOTUs. Taxonomic classification was performed with the SINA aligner (80) against SILVA (release 138.1) (81), RDP (release 11) (82), and GTDB (release 202) (83). Additionally, isolates sequences were submitted to BLASTn v.2.12.0+ (84) against a subset of the RDP database containing only cultured taxa (CCM) and another one containing only uncultured taxa (CEM) in order to test for their novelty. All ziOTUs that had less than 97% similarity with their CCM were considered as putatively novel strains. To assess the number of putative novel species and genera, the USEARCH software was used to cluster putative novel isolates to 97% and 94.5% similarity.

### Phylogenetic analyses

Two phylogenetic trees were constructed, one with all iOTUs to assess diversity of the complete collection, and another one only with putative novel ziOTUs to examine the phylogenetic placement of the novel strains. The general iOTUs tree was computed without reference sequences since it was only used to calculate phylogenetic diversity. For the tree with putative novel isolates, the closest sequence in SILVA Living Tree Project (LTP_12_2021) (81) for each ziOTU was inferred with BLASTn (84) and *Prochlorococcus marinus* subsp. *marinus* (ref AE017126 from LTP_12_2021) used as an outgroup. In both cases, the sequences were aligned with ClustalW in Geneious software v.2022.0.1 (77) and unaligned ends were trimmed. Phylogeny was constructed using maximum-likelihood inference with RAxML-NG 0.9.0 (85) and the GTR + G + I evolutionary model. For the iOTUs tree, bootstraps converged after 1,150 replications with 3% cutoff, while for the tree with putative novel isolates it did after 1,450 replications with 2% cutoff. The tree with putative novel isolates was plotted with iTOL v.6 (86).

### Compositional and statistical analyses

All analyses were carried out with the R software v.R 4.1.3 (87) and RStudio software v.1.3.1093 (88). Data manipulation was carried out mostly using packages tidyverse v.1.3.1 (89) and qdap v.2.4.3 (90), and plots were created in ggplot2 v.3.3.5 (91). An iOTU table was generated with 99%-clustered isolate 16S rRNA gene sequences. Normalized and rarefied tables were used in some of the subsequent analyses. To generate these rarefied tables, an iOTU table was first grouped by treatment and then rarefied to the lowest sampling effort (80 isolates in the DL treatment at t_0_) with 1,000 permutations with the package EcolUtils v.0.1 (92).

We determined whether culture media, season, and treatment shaped the composition of isolates at the class level. To compare iOTU composition between culture media, a proportional Venn diagram was plotted with package VennDiagram v.1.7.3 (93). To estimate sampling effort in each culture medium and season, rarefaction curves were performed with package vegan v.2.5.7 (94) using non-normalized iOTU tables. To estimate sampling effort across treatments, rarefaction curves were inferred with both non-normalized and rarefied iOTU tables. A PCoA based on Bray-Curtis distances followed by envfit analysis was carried out to test β-diversity across culture media, seasons, and treatments with package vegan v.2.5.7. α-diversity, richness, and the Chao1 estimator (95) were computed using the non-normalized iOTU table, while Shannon indices (96), FPD (97), and standardized effect size mean nearest taxon distance (98) were calculated with the normalized table. Packages ape v.5.6.2 (99) and picante v.1.8.2 (100) were used in order to infer FPD of the iOTUs tree. To test for differences between culture media, seasons, and treatments in all these indices, ANOVA tests and Tukey’s post hoc tests were carried out with package stats v.4.1.3. To test differences in culturability across culture media, Wilcoxon rank-sum test was performed with package stats after rejecting normal distribution with Shapiro-Wilk test from the same package. Welch’s *t* test was used to test significant differences in proportions of cultured reads between t_0_ and t_f_ of experiments (only when both samples had more than one replicate). Fisher’s exact test was performed to test whether some genera were more likely to be isolated in certain treatments using the non-normalized iOTU table and package stats v.4.1.3. When multiple testing, *P*-values were adjusted with the Benjamini-Hochberg FDR method (101). All statistical tests were made with the false-discovery rate set to 0.05.

### Comparison of isolates with ASVs

The 100%-clustered full 16S rRNA gene sequences of the isolates (ziOTUs) were compared to the ASVs defined from the V4-V5 region of the 16S rRNA gene using BLASTn v.2.12.0+ (84). Those ASVs that presented 100% similarity with one or more ziOTUs were considered as “cultured.” An ASV table was used to infer the rank and abundance of each cultured and uncultured taxa by treatment and season, at times t_0_ and t_f_. Additionally, the fraction of cultured taxa was calculated also for each treatment and season at times t_0_ and t_f_. The abundances used for these calculations were the means of all sample replicates. The closest neighbor to our isolates and ASVs was inferred searching our ziOTUs sequences against the NCBI 16S ribosomal RNA database (downloaded on 2022/03/01) using BLASTn. To calculate 16S amplicon sequencing abundances, corrected by their 16S rRNA gene copies, reads of each ASV were divided by their mean 16S rRNA gene copies by class except for SAR11, which was treated as a separate category. 16S rRNA gene copy numbers were obtained from rrnDB v.5.8 (67).

## Data Availability

All supplemental figures and tables can be found on GitHub (https://github.com/x-rv/Manuscript-2023). The 16S rRNA gene sequences of the isolates obtained in this study were deposited in GenBank under accession numbers OP342842 to OP344484. Amplicon sequencing data of the V4-V5 region of the 16S rRNA gene used in this study are publicly available in the European Nucleotide Archive under BioProject PRJEB60085.
